# Dress Nicer = Know More? Young Children’s Knowledge Attribution and Selective Learning Based on How Others Dress

**DOI:** 10.1371/journal.pone.0144424

**Published:** 2015-12-04

**Authors:** Kyla P. McDonald, Lili Ma

**Affiliations:** Department of Psychology, Ryerson University, Toronto, Ontario, Canada; Kyoto University, JAPAN

## Abstract

This research explored whether children judge the knowledge state of others and selectively learn novel information from them based on how they dress. The results indicated that 4- and 6-year-olds identified a formally dressed individual as more knowledgeable about new things in general than a casually dressed one (Study 1). Moreover, children displayed an overall preference to seek help from a formally dressed individual rather than a casually dressed one when learning about novel objects and animals (Study 2). These findings are discussed in relation to the halo effect, and may have important implications for child educators regarding how instructor dress might influence young students’ knowledge attribution and learning preferences.

## Introduction

Children rely heavily on what they are told by others to learn about the world, as knowledge about many domains is not accessible to their direct senses or experiences [1]. A great deal of research has demonstrated that when learning new information, young children consider a range of factors and display selective trust in one source over another. For example, preschoolers prefer to learn from previously reliable rather than unreliable informants [2]. They also prefer to learn from informants of their own gender, race, and spoken accent [3–5]. In addition, preschoolers favor information from speakers with more desirable traits such as honesty, benevolence, and intelligence [6, 7]. Relatively less is known about how potential informants’ physical appearance, such as clothing, would influence children’s knowledge attribution and selective learning from others. The goal of the present study is to shed light on this question.

Physical appearance is one of the major influences on our impression formation and social inferences [8]. In particular, ample empirical evidence has indicated that people automatically use the physical appearance of an individual to judge that person’s other traits such as intelligence, kindness, honesty, and trustworthiness [9–12]. This phenomenon is a clear example of the halo effect, a cognitive bias in which people use their overall feeling of an individual’s goodness or badness (i.e., global evaluations) to make judgments about that person’s specific traits [13, 14]. Of particular interest here is how two major elements of physical appearance—physical attractiveness and clothing—play a role in people’s trait attributions and learning experiences.

Many studies have provided support for the halo effect of physical attractiveness. For example, adults view physically attractive individuals as more knowledgeable, athletic, trustworthy, and friendly than unattractive ones, without being aware that physical attractiveness plays a role in their judgments [9–11]. This effect has also been found in educational settings. For instance, college students rate attractive instructors as more competent or having more expertise than unattractive ones [15–17]. Furthermore, as perceived instructors’ physical attractiveness increases, college students report greater motivation to learn and enhanced course satisfaction, feel that they learn better, and give higher teaching evaluations [17, 18]. Why does instructors’ physical attractiveness have such positive effects? One possible account is that attractiveness increases immediacy or the perception of closeness to people that are evaluated more positively, and that greater immediacy contributes to better learning experiences and outcomes [19, 20].

Research on the impact of clothing has obtained similar findings. In general, adults view individuals dressed casually such as wearing jeans and a t-shirt as less knowledgeable than those dressed more formally such as wearing a suit. In contrast, they view more formally dressed individuals as authority figures that resemble teachers and are more knowledgeable [21]. In addition, women tend to rate individuals in formal business clothing more positively on a range of traits such as intelligence, wealth, likeability, and trustworthiness [22]. Interestingly, clothing may also influence adults’ self-perception. For instance, formally dressed individuals are more likely to describe themselves using adjectives such as cultivated, restrained, and strategic, whereas those dressed casually are more likely to use adjectives such as easygoing, tolerant, and nonchalant [23].

Of particular relevance to the present study, the difference between formal and casual clothing may have important practical implications within the educational system. In classroom settings, more formally dressed instructors are often rated by college students as more knowledgeable about the subject matter, more competent, more friendly, and more organized and prepared than less formally or casually dressed ones [24–26]. However, there is evidence that the relationship between instructor dress and attributions of desirable traits may be moderated by the instructor’s age. For example, college students view older instructors that are casually dressed as more competent than younger instructors that are formally dressed [27]. Additionally, casually dressed graduate teaching assistants are perceived to have warmer attributes (e.g., sociability, extroversion) than formally dressed ones [28]. Nevertheless, the existing literature at large suggests that college students rate formally dressed instructors as generally more knowledgeable and competent than casually dressed ones. Furthermore, they prefer to learn and likely learn better from “well dressed” instructors [18].

Together, ample research has indicated that under the influence of the halo effect, adults pick up on the physical attractiveness and clothing of others to guide their trait attributions and learning preferences in classroom settings. Based on these findings, it has been recommended that instructors should make an effort to maintain a professional physical appearance in the classroom in order to exert positive influences on student learning [17, 18].

Does physical appearance have a similar impact on young children? This is an important question to address for at least two reasons. As mentioned earlier, children must rely on others to acquire knowledge about many aspects of the world. Research has shown that by 4 years of age, children are able to judge whether one person is more knowledgeable or a better source of information than another, based on epistemic grounds such as past reliability and expertise [2, 29–31]. In everyday interactions, however, children do not always have the option to reason with epistemic care. That is, children may not always have access to evidence about other people's previous reliability or expertise. In these circumstances, they may use their first impressions of an individual to make other judgments and guide their learning preferences, most likely based on physical appearance as it is a major influence on our impression formation [9–12]. In addition, physical appearance is modifiable, and thus may be more relevant to investigate for educational implications.

Developmental research has shown that the impact of physical attractiveness extends to young children. Like adults, children also make trait attributions based on how attractive an individual is, and that physical attractiveness has a significant effect on children’s ratings of an individual’s competence [10]. Moreover, it has been shown that when there is no evidence of informants’ epistemic reliability, preschoolers use the observed differences in their physical attractiveness to decide from whom to learn new knowledge. For example, when provided with conflicting labels for a novel object from a woman with a more attractive face versus a woman with a less attractive face, 4- and 5-year-olds prefer to endorse the label provided by the more attractive woman [32].

No studies to date have examined whether clothing may also influence young children’s trait attributions and learning preferences. Based on the effects of instructors’ formal versus casual clothing on college students [24–26] as well as children’s attention to physical attractiveness in trait attributions [10] and selective social learning [32], it is conceivable that children would attribute greater general knowledge to and prefer to seek novel information from a formally dressed individual rather than a casually dressed one. The goal of the present study was to examine these possibilities. Across two studies, a formally dressed individual was pitted against a casually dressed one. In Study 1, children were asked which individual they thought was more knowledgeable about new things in general (i.e., knowledge attribution). In Study 2, children were asked to decide from whom they would like to seek information about the names of novel objects and animals (i.e., selective social learning).

Four- and 6-year-olds were tested in both studies. Four-year-olds were chosen based on previous findings that the ability to systematically consider various cues (e.g., past reliability, desirable traits) in selective social learning is robustly observed in 4-year-olds, but not always in 3-year-olds [29, 30, 33, 34], and that by 4 to 5 years of age, children display learning preferences based on informants’ physical attractiveness [32]. Six-year-olds were also tested for possible developmental comparisons: Relative to 4-year-olds, most 6-year-olds in Canada have experienced at least one full year within the formal school system (i.e., Kindergarten or Grade 1) [35], and may have different perceptions or expectations about instructors’ clothing in relation to their teaching competence. Thus, 6-year-olds might show a greater sensitivity to an individual’s clothing in the context of knowledge attribution and social learning than 4-year-olds do.

In addition to the possible age difference, we were also interested in whether the impact of clothing on children’s knowledge attribution and social learning would be the same for different racial groups. As such, in both studies, children were presented with either a pair of White individuals (2 trials) or a pair of East Asian individuals (2 trials) that differed in clothing. First, our participants were recruited from a metropolitan area in Canada that is highly diverse and multicultural, where East Asians constitute one of the largest visible minority groups [36]. Therefore, it would be ecologically more valid to include both White trials and East Asian trials. Second, there is evidence that people stereotype East Asian individuals as more intelligent than individuals of other ethnicities in certain academic areas [37, 38]. Based on this finding, it is possible that children might perceive the East Asian pair as equally good sources of knowledge regardless of their clothing. With these two considerations in mind, we included trial type (White vs. East Asian) as a within-subjects factor for exploratory purposes.

## Study 1

In this study, 4- and 6-year-olds viewed photos of two female adults and were asked whom they thought knew more about novel things in general. The two individuals were of the same race (White or East Asian) but differed in how they were dressed (formally vs. casually). Previous studies have indicated that preschoolers are subject to the halo effect in their social inferences about other people [39–41]. Based on these studies and the relevant findings with adults as reviewed earlier [25, 26], we predicted that children would be more likely to identify the formally dressed individual as more knowledgeable than the casually dressed one. It was also expected that the influence of clothing would be stronger on 6-year-olds than on 4-year-olds. We included two trial types (White vs. East Asian) for exploratory purposes, and had no clear predictions about their effects on children’s responses.

### Method

#### Participants

The final sample included 32 children, including 16 four-year-olds (*M* = 52.5 months, range = 48.2–59.2 months; 8 girls) and 16 six-year-olds (*M* = 77.4 months, range = 72.3–82.7 months; 8 girls). Half of the children were White (16), with some Asian (8) and some Other Race (8). One additional child was excluded from the final sample due to language barriers. Children in this study and in Study 2 were recruited from a metropolitan area in Canada through a participant database, several childcare programs, and a science center.

The Research Ethics Board at Ryerson University approved the use of human subjects for this research. Written consent was obtained from the parents or legal guardians of the participants. Each participant verbally agreed to take part in the study.

#### Materials and stimuli

Eight female adults were photographed for the stimuli, 4 White and 4 East Asian. Two color photos were taken of each individual (16 photos in total). In one photo, the individual was dressed formally, wearing a black, brown, or grey blazer with a dress shirt and black dress pants. In the other photo, she was dressed casually, wearing a black, brown, or white t-shirt with blue jeans. In both photos, the individual sat in the same position, with her legs crossed, and her hands on a chair with an upright posture.

Five PowerPoint slides were used to present the stimuli on a laptop computer. They were shown to the child in one of 8 randomized orders. The first slide introduced the task and displayed images of various novel animals and objects. The remaining 4 slides were used for the 4 test trials, one slide per trial. Each slide displayed the photos of two individuals (left or right side counterbalanced), one formally dressed and one casually dressed.

On each trial, clothing was counterbalanced across participants (i.e., each individual appeared formally dressed for half of the participants, and casually dressed for the other half of the participants). Therefore, a total of 8 pairings of individuals were used during the test phase. Within each pair, the two individuals were presented side by side. They differed in clothing as described above, but were matched in terms of race, age, skin color, hair color, hairstyle, and facial expressions.

#### Design and procedure

This study used a 2 (trial type: White vs. East Asian) x 2 (age: 4 vs. 6 years) mixed design, with trial type as the within-subjects factor and age as the between-subjects factor. During the study, the child sat directly in front of the laptop computer, and a female researcher sat beside him or her. The parent either stayed in another room or sat behind the child, and was told not to interfere with the procedure. The procedure involved two phases as detailed below.

**Introduction phase**: Pointing to the images on the introduction slide, the researcher said to the child, “There are so many things around the world that it is hard to know the names of them all!” The test phase with 4 trials then followed.

**Test phase**: On each of the 4 test trials, the researcher asked the child to indicate which of the two individuals they thought was more knowledgeable, “Who do you think knows more about new things, this person (pointing left) or this person (pointing right)?” The child was allowed to make a choice either verbally or by pointing.

#### Coding and reliability

The researcher coded children’s responses during the study. For reliability, a trained research assistant coded 50% of the sample from video recordings. The two raters had no disagreement.

### Results and Discussion

Preliminary analyses indicated that there was no significant effect of presentation order, the child’s gender, or the child’s race on children’s performance in this study and in Study 2. Therefore, these variables were not included in the main analyses reported below. All reported *p* values are two-tailed.

Each child received a choice score for each trial type, by calculating the proportion of trials (out of 2) on which he or she thought the formally dressed individual was more knowledgeable about new things (i.e., the dependent variable). These choice scores were categorical with a multinomial distribution. Based on the recommendation of Jaeger (2008) [42], they were submitted to a repeated-measures Generalized Linear Model with Generalized Estimating Equations (GEE), in which trial type was the within-subjects factor and age was the between-subjects factor. The results indicated that there was no significant main effect of trial type or age, Wald *χ*² (1) = 0.05, *p* = .94, and Wald *χ*² (1) = 0.86, *p* = .35, respectively. In other words, children responded similarly across trial type and age. No significant interaction was found, Wald *χ*² (1) = 1.12, *p* = .29.

**Fig 1:**
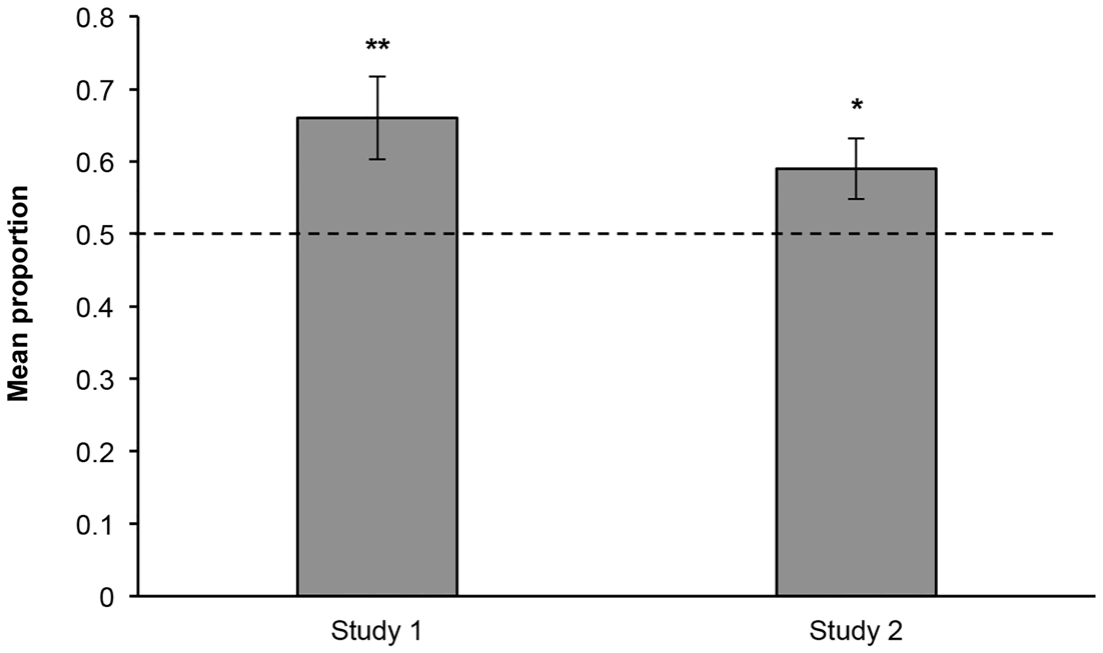
Mean Proportion of Trials Choosing the Formally Dressed Individual Across Studies. **p = .01; *p = .03, as compared to chance (.50).

We then compared children’s overall performance to chance responding (.50). Since the main effects and the interaction were not significant, we collapsed data across trial type and age. In general, children judged the formally dressed individual as more knowledgeable on about two thirds of the trials (*M* = .66, *SD* = .322), which is significantly more often than would be expected by chance, *t* (31) = 2.74, *p* = .01, Cohen’s *d* = 0.50 (Fig 1).

Thus, 4- and 6-year-olds attributed general knowledge to unfamiliar others based on their clothing, and there was no significant age difference. Overall, children viewed a formally dressed individual as more knowledgeable about new things in general than a casually dressed one. This finding is consistent with adult literature suggesting that professional attire may symbolize greater knowledge and competence [18, 25, 43]. The main effect of trial type was not significant, indicating that children used clothing as a cue to the knowledge state of unfamiliar others that were either White or East Asian.

Note that on each test trial, each of the two individuals appeared formally dressed for half of the participants, and casually dressed for the other half of the participants. Nevertheless, some differences existed in the two individuals’ physical attractiveness and clothing color. Twenty adults (*M* = 28 years; 10 females) rated each individual’s physical attractiveness both when she was formally dressed and when she was casually dressed, on a 5-point Likert scale ranging from 1 (very unattractive) to 5 (very attractive). The ratings differed significantly for all 8 pairings (*p* < .05). With respect to clothing color, we used neutral colors so that the two individuals’ outfits were not likely to be visually appealing one way or another. On 5 of the 8 pairings, each pair of individuals wore tops of a matching color (and black dress pants or blue jeans). On the remaining 3 pairings, each pair of individuals wore tops of different colors (e.g., a white t-shirt vs. a black blazer). Despite these differences in the two individuals’ physical attractiveness and clothing color, during the study, children were biased toward the professionally dressed individual (regardless of her perceived level of attractiveness or clothing color) on 7 pairings; on the one remaining pairing, children were biased toward the casually dressed individual who was rated as less attractive than the professionally dressed one, and the pair wore tops of a matching color. Therefore, children’s responses were systematically based on how the individuals dressed, not based on their physical attractiveness or clothing color.

About 53% of the children (17/32) provided explanations for their choices spontaneously, or were asked informally by the researcher at the end of the study. Although several children responded with “I don’t know” or hard-to-interpret explanations (e.g., “because she plays piano and likes animals”), some children did provide informative justifications for why they chose the formally dressed individual as more knowledgeable, such as “she worked”, “she looks like a teacher”, “she looks bigger, has grown up clothes on, and has a job”, and “she looked older”. These “occupation” or “age” related explanations fall in line with the literature. For instance, adults often infer an individual’s occupational role and status from his or her uniform or clothing [44]. Also, college students view older instructors as more competent than younger ones [27], and children also view age as an important predictor of another person’s competence [45].

To summarize, Study 1 showed that 4- and 6-year-olds identified a formally dressed individual as more knowledgeable about new things in general than a casually dressed one. It remains an open question whether informants’ clothing has a similar impact on children’s actual learning preferences, for example, deciding from whom to inquire about novel objects and animals. Study 2 was conducted to address this question, in which 4- and 6-year-olds were tested. Note that the two studies addressed two separate questions, and we were interested in whether knowledge attribution based on how others dress can be generalized to a different context (i.e., selective social learning), which required that the age groups in the two studies be comparable.

## Study 2

In this study, 4- and 6-year-olds were asked to indicate from whom, a formally dressed individual or a casually dressed one, they would like to inquire about the names of novel objects and animals. We predicted that if children use potential informants’ clothing to guide their information seeking, their responses would reveal one of three possible patterns. One possibility was that based on the results of Study 1, children would prefer to seek novel information from the formally dressed individual rather than the casually dressed one, regardless of the knowledge domain. Alternatively, based on the findings that casually dressed instructors can be associated with warmer characteristics such as friendliness [28], and that children prefer to learn from benevolent informants rather than mean ones [6, 29], it is also possible that children would prefer to ask the casually dressed individual for novel information. In addition, children’s responses might depend on the knowledge domain, in that they would prefer to ask the casually dressed individual when learning about novel animal names, based on a possible belief that someone dressed casually as opposed to formally would be more likely to be around animals and thus have more domain knowledge about animals.

Study 1 showed that the effects of age and trial type (White vs. East Asian) on children’s knowledge attribution were not significant. Nevertheless, in Study 2 we chose to include these two factors as it is unclear whether their non-significant effects on knowledge attribution would be generalized to children’s selective information seeking.

### Method

#### Participants

Participants were 66 children, including 33 four-year-olds (*M* = 54.1 months, range = 48.3–59.7 months; 22 girls) and 33 six-year-olds (*M* = 77.6 months, range = 72.3.– 83.7 months; 15 girls). Thirty-four children participated in the Object condition, and 32 in the Animal condition. About half of the children were White (30), with some Asian (22) and some Other Race (14). Two additional children were excluded from the final sample because of parental interference (1) or an experimenter error (1).

#### Materials and stimuli

The same materials from Study 1 were used, with an addition of the color photos of 4 novel objects (a baby teething tool, a light-bulb changer, a vacuum cleaner, and a citrus juice reamer) and 4 novel animals (a galah, a bilby, a dugong, and a civet).

A total of 9 PowerPoint slides were used to present the stimuli on a laptop computer. The first slide introduced the task and displayed the 4 novel objects or animals mentioned above. Then two slides were used for each of the 4 test trials: The first slide displayed the image of a novel object or animal; the second slide displayed the contrast between a formally dressed individual and a casually dressed one as in Study 1, with the addition of the object or animal image directly below them in the middle. The counterbalancing was conducted in the same way as in Study 1.

#### Design and procedure

A mixed design was used, with trial type (White vs. East Asian) as the within-subjects factor and condition (object vs. animal) and age (4 vs. 6 years) as the between-subjects factors. The experimental setting was the same as in Study 1.

The procedure also involved two phases: introduction and test. The introduction phase was conducted in the same manner as in Study 1. During the test phase, on each test trial, the researcher first showed the slide displaying the novel object or animal and said to the child, “Look at this new object/animal. Let’s find out what it is called!” She then showed the second slide and said to the child, “I bet one of them knows better and can help us! Who should we ask to find out the name of this object/animal? Should we ask her (pointing to left), or should we ask her (pointing to right)?” At the end of the study, the researcher debriefed the child about the name of each novel object or animal.

#### Coding and reliability

Initial coding and reliability coding were conducted in the same way as in Study 1. There was no disagreement between the two raters.

### Results and Discussion

As in Study 1, each child received a choice score for each trial type (i.e., the proportion of trials choosing the formally dressed individual). A repeated-measures Generalized Linear Model with GEE revealed no significant main effect of trial type, condition, or age on children’s choice scores, Wald *χ*² (1) = 0.76, *p* = .38, Wald *χ*² (1) = 0.39, *p* = .53, and Wald *χ*² (1) = 0.30, *p* = .59, respectively. No significant two-way interaction was found between trial type and condition, trial type and age, or condition and age, Wald *χ*² (1) = 0.03, *p* = .86, Wald *χ*² (1) = 0.05, *p* = .83, Wald *χ*² (1) = 1.0, *p* = .32, respectively.

Interestingly, there was a significant three-way interaction between trial type, condition, and age, Wald *χ*² (1) = 5.05, *p* = .025, Cramer’s *V* = .28. Separate repeated-measures Generalized Linear Model with GEE was then conducted for each age group. For 4-year-olds, no significant main effect of trial type or condition was observed, Wald *χ*² (1) = 1.31, *p* = .25, Wald *χ*² (1) = 0.21, *p* = .64, respectively. The interaction between trial type and condition approached significance, Wald *χ*² (1) = 2.92, *p* = .09. For 6-year-olds, no significant main effect or interaction emerged.

Next, we compared children’s performance to chance expectation (.50). Data were collapsed across trial type, condition, and age, as there were no significant main effects or robust interactions. Overall, children chose to ask the formally dressed individual significantly more often than would be expected by chance (*M* = .59, *SD* = .341), *t* (65) = 2.16, *p* = .034, Cohen’s *d* = 0.27 (Fig 1).

To summarize, Study 2 extended the findings of Study 1 and showed that when learning about the names of novel objects and animals, 4- and 6-year-olds had an overall preference to seek information from a formally dressed individual rather than a casually dressed one. That is, consistent with the adult literature, the difference between formal and casual clothing plays a role in children’s trait attributions and learning preferences.

## General Discussion

The present data indicated that 4- and 6-year-olds perceived a formally dressed individual to be more knowledgeable about new things than a casually dressed one. Furthermore, children had an overall preference to seek help from a formally dressed individual rather than a casually dressed one when learning about the names of novel objects and animals. These main findings are discussed in turn.

Previous studies have indicated that adults generally perceive formally dressed individuals to be more competent and knowledgeable than casually dressed ones, and that this effect has also been found in classroom settings [25–27, 43]. In line with this literature, 4- and 6-year-olds in Study 1 identified a formally dressed individual as more knowledgeable about new things in general than a casually dressed one. This attribution of general knowledge based on clothing may be explained by the halo effect [13]. That is, when children had limited background information about the unfamiliar individuals, they used apparent differences in physical appearance, such as the difference between formal and casual clothing, to guide their judgments of other traits (e.g., knowledge state) of these individuals.

Consistent with this explanation, previous studies have demonstrated that young children are subject to the halo effect when they make social inferences about other people. For example, 4- and 5-year-olds give more positive evaluations such as “smart” and “athletic” to a nice individual than to a mean one [40, 41]. Also, 5-year-olds predict a previously accurate individual to be more prosocial than a previously inaccurate one [39]. In addition, 3- to 10-year-olds in South Africa associated higher levels of wealth more often with White than with Black people [46].

In the present study, two possible mechanisms, separately or in combination, might have mediated the halo effect of clothing on children’s knowledge attribution. First, clothing can serve as a cue for an individual’s occupational or social status [44, 47]. It may therefore activate stereotypes associated with the perceived occupation or authority of that individual. For example, adults perceive individuals dressed more formally as authority figures that resemble teachers and are more knowledgeable than casually dressed ones [21]. In line with this possibility, some children in Study 1 explained that they thought the formally dressed individual was more knowledgeable because “she worked” or “she looked like a teacher”.

The second possible mechanism may have something to do with children’s inferences about an individual’s age from his or her clothing. It has been shown that 4- and 5-year-olds recognize that adults are generally more knowledgeable about the world than children [48, 49]. In light of this finding, in Study 1 children might have perceived the formally dressed individuals to be older and thus have greater knowledge. Consistent with this possibility, some children judged the formally dressed individual as more knowledgeable about new things because “she looked older”. Note that these two possibilities were derived from some children’s informal explanations for their choices of the formally dressed individuals, and thus await more systematic examination in future research.

Study 2 provided further evidence that 4- and 6-year-olds not only attributed greater general knowledge to formally dressed individuals, but also preferred to seek information from them when learning about novel objects and animals. As previously mentioned, there is evidence that college students prefer to learn and likely learn more effectively from “well-dressed” instructors [18]. The present data extend this finding and provide the first indication that at 4 to 6 years of age, children might also attend to how others dress in their selective information seeking, which is consistent with the work by Bascandziev and Harris (2014) [32], in that children might selectively learn from others based on non-epistemic grounds. That is, when there is no good reason (e.g., past accuracy) to infer the epistemic reliability of potential informants, children pick up on their physical appearance such as clothing to decide from whom to learn novel information.

Findings from this research may contribute to our understanding of how children perceive others as sources of information, an important aspect of early social cognition. Young children are generally more credulous than adults or older children, to the point that sometimes they accept obviously false information that contradicts their firsthand observations [50]. In order to avoid learning inaccurate information from others, young children must take a critical stance and place greater trust in more reliable sources [51]. Such critical thinking is linked to the development of certain social-cognitive skills, such as theory of mind [51–54] and executive functions [55]. In everyday interactions, however, children do not always have access to evidence about another person’s epistemic reliability to make a well thought-out judgment or decision based on critical thinking. In these situations, they may turn to observable differences in physical appearance, such as how others dress, to judge the knowledge state of others and decide whether one person is a better source of information than another (i.e., the halo effect). On the one hand, this heuristic may work well under some circumstances for young children who do not have adequate social-cognitive skills or sufficient information to critically analyze the knowledge state and reliability of an informant. On the other hand, this heuristic can lead to unwanted consequences, in that young children may be more prone to make stereotypical judgments about others based on how they dress or accept false information from someone who is well dressed.

The present findings may also have important implications for early educators and administrators looking for ways to benefit student learning. According to some researchers, physical appearance is a non-instructional factor that influences students’ learning preferences and how well they retain new information, as demonstrated by empirical evidence with adults [18]. As reviewed earlier, physical appeal increases immediacy, and greater immediacy contributes to better learning experiences and outcomes [19, 20]. Thus, if children attribute greater knowledge to and prefer to learn from formally dressed individuals, through increased immediacy, they might actually learn more effectively from formally rather than casually dressed instructors. This may be due to several factors, including a greater motivation to learn from, more sustained attention to, and an increased faith in the information presented by formally dressed instructors [17, 18]. As such, instructor dress may play an important role in supporting young children’s learning in the classroom. To address this possibility, future work can directly compare formally dressed instructors with casually dressed ones and examine whether young students attribute greater general knowledge to the former and prefer to learn from them in classroom settings.

The above-mentioned implications are based on the assumption that in the absence of epistemic cues, children view formally dressed instructors as better transmitters of knowledge than casually dressed ones, which might be incongruent with student-centered pedagogy. Derived from the constructivist position that children actively construct their own understanding and knowledge of the world through experimentation and discovery [56, 57], student-centered pedagogy requires instructors to adapt to the role of facilitators and promote student autonomy in learning through guided participation [58]. Influencing learning preferences through instructor dress, however, is premised on children’s tendency to seek information from formally dressed instructors that they perceive to be better at “teaching” or “giving” them knowledge, which might be inconsistent with the student-centered approach to cultivating children’s autonomous learning behaviors such as independent problem-solving and actively constructing their own knowledge instead of passively accepting the information given by instructors. Thus, although formal instructor dress may contribute to better learning experiences and outcomes [18], instructors should go beyond maintaining a professional physical appearance and engage in teaching practices that can help them foster students’ autonomous learning.

While children may have favorable views of formally dressed instructors as knowledge transmitters, when epistemic cues such as expertise or past reliability are available, they might choose to learn from instructors who are experts in the knowledge domain or have been a reliable source in the past, regardless of whether they dress formally or causally. This possibility awaits future examination. Another path for future research concerns how children selectively learn from people that are dressed in ways other than “casually” or “formally”. For example, how do children react to potential informants dressed in “athletic” or “sciencey” clothing as opposed to other attire? Presumably, children might display selective learning based on inferences made from clothing about informant domain expertise (e.g., learning about fitness from an individual wearing athletic clothing). Supporting this, studies have show that preschoolers are aware that people may have different levels of expertise across different domains [59], and that preschoolers prefer to learn about one domain (e.g., dogs) from individuals with relevant rather than irrelevant expertise (e.g., from dog experts as opposed to artifact experts) [60].

To conclude, the present data indicate that under the possible influence of the halo effect, 4- and 6-year-olds favor formally dressed individuals over casually dressed ones in both knowledge attribution and selective social learning. These findings have important implications within the educational system, suggesting the possibility that in classroom settings, instructors who dress themselves more formally and thus maintain a professional physical appearance may be more likely to exert positive influences on young students’ learning experiences and outcomes.

## Supporting Information

S1 Fig Data for Fig 1. (XLSX)

S1 File Data for Studies 1 and 2. Data used for the Generalized Linear Models with Generalized Estimating Equations (GEE); one data sheet per study.
(XLSX)
